# Hepatic Duct Perforation Following Mitral Valve Replacement for Infectious Endocarditis: A Case Report

**DOI:** 10.7759/cureus.108007

**Published:** 2026-04-30

**Authors:** Tomoki Hongo, Yuka Mishima, Hiroaki Ono, Kiyotoshi Oishi, Kenji Wakabayashi

**Affiliations:** 1 Department of Emergency Medicine and General Internal Medicine, Fujita Health University Hospital, Toyoake, JPN; 2 Center for Clinical Bioethics, Georgetown University Medical Center, Washington, DC, USA; 3 Department of Intensive Care Medicine, Institute of Science Tokyo, Tokyo, JPN; 4 Department of Hepato-Biliary-Pancreatic Surgery, Saitama Medical University International Medical Center, Hidaka, JPN; 5 Department of Cardiovascular Surgery, Institute of Science Tokyo, Tokyo, JPN

**Keywords:** bile leakage, clinical case report, conservative treatment, hepatic duct perforation, intra-abdominal abscess, mitral valve replacement, postoperative complication

## Abstract

Hepatic duct perforation (HDP) is a rare and life-threatening condition in adults, typically caused by direct injury or increases in intraductal pressure. The risk of HDP following cardiac surgery is poorly documented, although predisposing factors, such as bilirubin overload and ischemia from cardiopulmonary bypass, share common pathophysiological mechanisms with postoperative cholecystitis. We report the case of an 80-year-old woman who underwent mitral valve replacement for infectious endocarditis. Preoperatively, she was asymptomatic with mild common hepatic duct dilatation but no stones or ascites. However, her condition was also complicated by anemia and sepsis-induced disseminated intravascular coagulation. The cardiac surgery involved 153 minutes of extracorporeal circulation and required massive transfusion. On postoperative day (POD) four, she developed respiratory failure requiring re-intubation. A subsequent computed tomography and abdominal paracentesis confirmed HDP. Given her hemodynamic instability and prohibitive surgical risk, percutaneous drainage was performed. Although the acute biliary peritonitis initially improved, she developed persistent low-grade inflammation. On POD 61, she suffered from pancytopenia and reactivation of both varicella-zoster virus and cytomegalovirus, ultimately leading to multiple organ failure and death on POD 84. HDP can be a critical postoperative complication of cardiac surgery, potentially triggered by bypass-induced hypoperfusion and systemic stress. Despite successful percutaneous drainage, the persistent inflammatory state may induce secondary immunodeficiency and fatal opportunistic viral reactivation. This case highlights the inherent limitations of conservative management and the difficulty of deciding on definitive surgical intervention in critically ill postoperative patients.

## Introduction

Hepatic duct perforation (HDP) is a rare and highly fatal condition in adults, typically caused by increased intraluminal pressure due to distal obstruction or direct injury to the duct wall [1]. In the context of cardiac surgery, abdominal complications impact postoperative mortality [2]. Among these, postoperative cholecystitis is a known complication following cardiac surgery due to bilirubin overload, cholestasis, and ischemic injury caused by cardiopulmonary bypass and increased bleeding [3]. Although these mechanisms align with known etiologies of HDP, the potential risk of HDP is not well established in postcardiac surgery.

The most common approach to biliary perforation is surgical intervention, such as drainage, cholecystectomy, and T-tube placement, for definitive management of both the perforation and associated peritonitis [4,5]. Nonetheless, in the hemodynamically unstable environment following cardiac surgery, physicians may face a clinical dilemma between the necessity of surgical source control and the risks of re-operation. This case report highlighted the limitations of conservative management for HDP and the difficulty of timely definitive intervention, especially in critically ill patients.

The article was previously presented as a meeting abstract at the 51st Annual Meeting of the Japanese Society of Intensive Care Medicine on March 14, 2024.

## Case presentation

An 80-year-old woman presented to a referring hospital with fever, malaise, and difficulty in mobilization. Two sets of blood cultures grew methicillin-susceptible *Staphylococcus aureus* (MSSA), and transthoracic echocardiography revealed vegetations on the mitral valve, leading to a diagnosis of infective endocarditis (IE). Because multidisciplinary management, including surgical intervention, was required, she was transferred to our hospital. Physical examination on admission was remarkable only for a pansystolic murmur at the apex. Notably, she had no abdominal pain or jaundice, and laboratory tests did not show any elevation of hepatobiliary enzymes. An elevated white blood cell count, anemia, and thrombocytopenia due to disseminated intravascular coagulation (DIC) were observed in association with IE (Table 1). Preoperative contrast-enhanced computed tomography (CT) demonstrated mild dilatation of the common hepatic duct without gallstones, common bile duct stones, ascites, or any history suggestive of prior biliary disease.

**Table 1:** Laboratory investigations on admission, postoperative days one and four POD: postoperative day

Parameter (unit)	Reference range	On admission	POD 1	POD 4 (re-intubation)
White blood cell count (×10^9^/L)	3.3-8.6	25.7	19.2	28.6
Hemoglobin (g/dL)	13.7-16.8	10.3	11.8	13.8
Platelet count (×10^9^/L)	158-348	23	123	68
Prothrombin time activity (%)	70-130	102.9	115.3	84.4
Prothrombin time–international normalized ratio	0.9-1.1	0.99	0.93	1.08
Activated partial thromboplastin time (seconds)	24-38	39.2	25.9	33.9
Fibrin degradation products (µg/mL)	<5	202.6	-	-
Albumin (g/dL)	4.1-5.1	1.9	3.6	3
Blood urea nitrogen (mg/dL)	8-20	57.6	47.8	59.5
Creatinine (mg/dL)	0.6-1.1	0.87	0.99	1.14
Aspartate aminotransferase (U/L)	13-30	21	62	44
Alanine aminotransferase (U/L)	7-23	17	27	12
Gamma-glutamyl transpeptidase (U/L)	13-64	29	54	67
Total bilirubin (mg/dL)	0.4-1.5	0.8	1.5	1
Creatine kinase (U/L)	59-248	46	566	117
Amylase (U/L)	44-132	579	323	86
Glycated hemoglobin (%)	4.6-6.2	6.3	-	-

She underwent minimally invasive cardiac surgery for mitral valve replacement. The procedure lasted 267 minutes with 153 minutes of extracorporeal circulation, 544 mL of blood loss, and transfusions of 12 units of packed red blood cells, 16 units of fresh frozen plasma, and 60 units of platelet concentrate. These were considered to be due to DIC-related coagulopathy.

**Figure 1:**
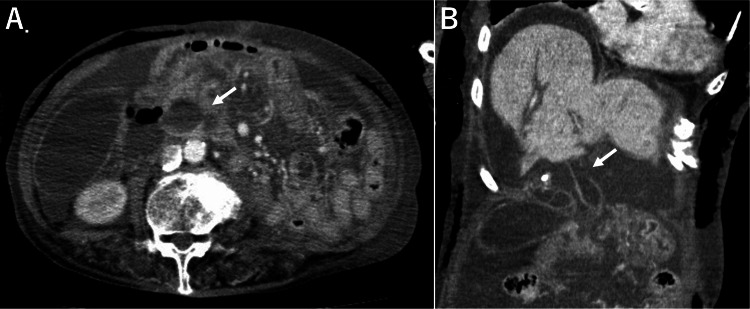
Contrast-enhanced CT on POD 4 showing hepatic duct perforation (A) Dilated common hepatic duct (arrow). (B) A focal contrast defect was observed in the wall of the common hepatic duct (arrow), suggestive of the perforation site. CT: computed tomography; POD: postoperative day

Postoperatively, she developed systemic inflammation and respiratory failure requiring re-intubation on postoperative day (POD) 4. Despite the lack of localized abdominal symptoms, contrast-enhanced CT revealed new-onset ascites, worsened biliary dilatation without stones, and a contrast defect in the common hepatic duct (Figure 1). Subsequent abdominal paracentesis and a trans-drain contrast study confirmed biliary peritonitis resulting from HDP.

**Figure 2:**
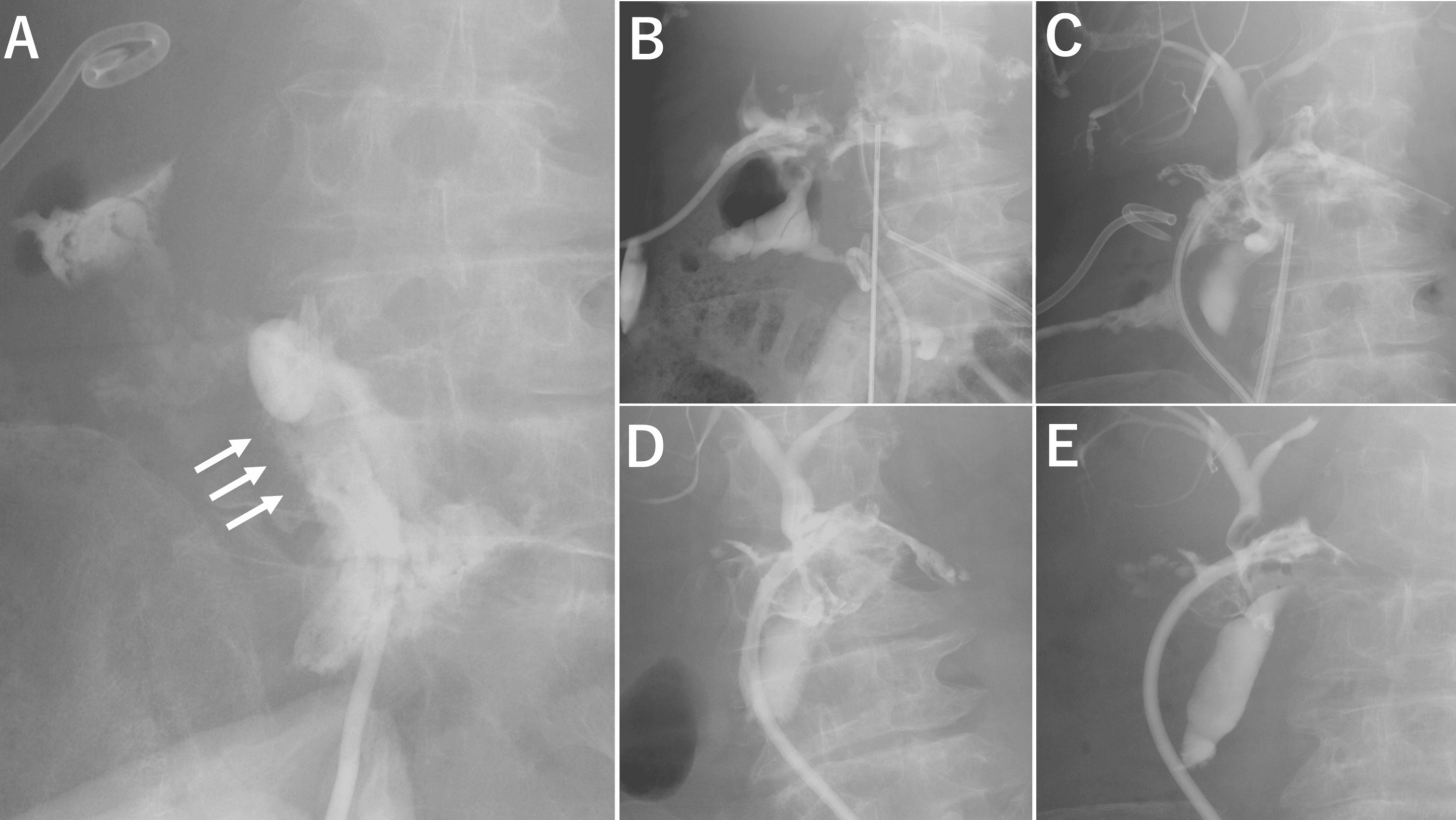
Serial drainage study demonstrating abscess regression (A) Tubography on POD 7 indicating the opacified hepatic duct (arrows). (B)-(E): Sequential contrast studies showing consistent shrinkage of the abscess cavity on POD 7 (B), POD 21 (C), POD 35 (D), and POD 48 (E). POD: postoperative day

Ascitic fluid culture grew *Enterococcus faecium* and *Candida albicans*. Empiric antimicrobial therapy was initiated with cefepime, vancomycin, metronidazole, and fluconazole. Surgical treatment was deemed high-risk due to her severe general condition; thus, percutaneous abdominal drainage was performed. Although blood culture obtained on POD 28 yielded *Enterococcus faecium*, which turned negative on POD 35. The abscess gradually decreased (Figure 2).

**Figure 3:**
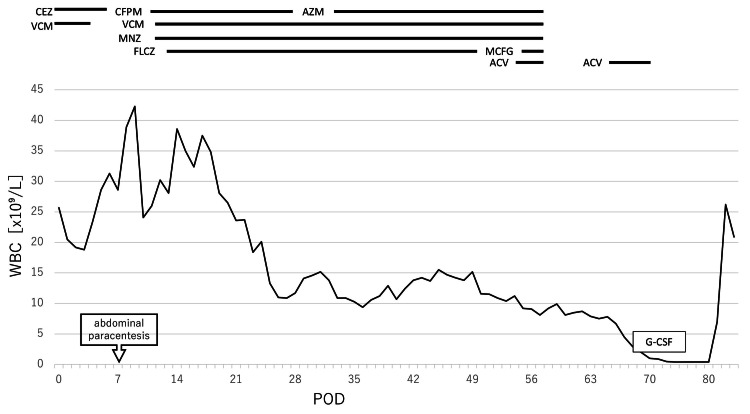
Clinical course showing trends in white blood cell count and the duration of antimicrobial and antiviral therapies ACV: acyclovir; AZM: azithromycin; CEZ: cefazolin; CFPM: cefepime; FLCZ: fluconazole; G-CSF: granulocyte colony-stimulating factor; MCFG: micafungin; MNZ: metronidazole; POD: postoperative day; VCM: vancomycin; WBC: white blood cell Image was created using Microsoft Excel and Microsoft PowerPoint (Microsoft Corporation, Redmond, WA).

On the other hand, while inflammation markers initially improved, mild inflammation persisted (Figure 3). On POD 41, she developed herpes zoster and received acyclovir. On POD 61, she developed pancytopenia, and reactivation of both varicella-zoster virus and cytomegalovirus was confirmed. Her condition deteriorated, progressing to multiple organ failure, including liver failure, and ultimately death on POD 84.

## Discussion

This case report presents two key clinical observations. The first is the potential association between HDP and cardiac surgery. The second is the demonstration of the critical limitations of conservative management for HDP in a critically ill patient, leading to prolonged critical illness and fatal opportunistic viral reactivation.

In our case, no biliary diseases were identified on preoperative CT, suggesting that HDP developed through a multifactorial mechanism. First, the patient's preoperative state was severely compromised by anemia and sepsis-induced DIC, which reduced the oxygen delivery and caused baseline microcirculatory impairment. Second, exposure to cardiopulmonary bypass is a known risk factor for organ ischemia [2]. While the bypass time in this case was not exceptionally long, vascular fragility due to advanced age likely further increased the risk of ischemia, which is a contributing factor to HDP [1]. In addition, the massive transfusion required suggested extreme systemic stress and potential bilirubin overload, leading to cholestasis [1,3] and further weakening the already ischemic ductal wall. The fact that the intra-abdominal isolates differed from the causative agent of IE (MSSA) supported the hypothesis that HDP was a secondary complication of surgical stress rather than a primary infectious focus. We emphasize that, in high-risk patients, the cumulative effect of anemia, DIC, bypass-induced hypoperfusion, and massive transfusion can reach a pathological threshold leading to HDP, mirroring the pathophysiology of postoperative cholecystitis [3].

Regarding management, surgical intervention remains the gold standard as it allows for definitive source control [4,6]. However, the early symptoms of HDP are non-specific [1], which can be masked by the sedation and systemic inflammation common in the intensive care unit (ICU). By the time HDP was confirmed, our patient had progressed to multi-organ failure, creating a profound clinical dilemma: the necessity of definitive surgery versus the high risk of operative mortality. Although percutaneous drainage initially controlled the acute peritonitis, the subsequent persisting chronic inflammation likely induced "persistent inflammation, immunosuppression, and catabolism syndrome" [7]. This syndrome-driven secondary immunodeficiency could precipitate opportunistic viral infections, followed by multi-organ failure [8]. This sequence underscores that conservative management alone may be inadequate for achieving long-term survival in high-risk patients. Furthermore, it emphasizes the clinical dilemma of recognizing the importance and difficulty of making a timely decision for definitive surgical management, even when initial conservative measures appear successful.

This case report had several inherent limitations. First, as a single case, the hypothesized association between specific cardiac surgical factors and HDP remains suggestive. Second, due to the patient's hemodynamic instability, we cannot definitively conclude whether early surgery would have altered the outcome.

## Conclusions

HDP can be a rare but critical postoperative complication of cardiac surgery. This case underscores that, even when local control of biliary peritonitis is achieved, the resulting prolonged inflammatory course can induce immunosuppression and fatal viral reactivation. Therefore, clinicians must maintain a high index of suspicion for HDP in patients with unexplained postoperative inflammation and remain vigilant for the subsequent risk of immunological failure during prolonged ICU stays.
